# Embryos in the Fast Lane: High-Temperature Heart Rates of Turtles Decline After Hatching

**DOI:** 10.1371/journal.pone.0009557

**Published:** 2010-03-10

**Authors:** Wei-Guo Du, Bo Zhao, Richard Shine

**Affiliations:** 1 College of Biological and Environmental Sciences, Hangzhou Normal University, Hangzhou, Zhejiang, People's Republic of China; 2 School of Biological Sciences, University of Sydney, Sydney, New South Wales, Australia; University of Queensland, Australia

## Abstract

In ectotherms such as turtles, the relationship between cardiovascular function and temperature may be subject to different selective pressures in different life-history stages. Because embryos benefit by developing as rapidly as possible, and can “afford” to expend energy to do so (because they have access to the yolk for nutrition), they benefit from rapid heart (and thus, developmental) rates. In contrast, hatchlings do not have a guaranteed food supply, and maximal growth rates may not enhance fitness—and so, we might expect a lower heart rate, especially at high temperatures where metabolic costs are greatest. Our data on two species of emydid turtles, *Chrysemys picta, *and *Graptemys pseudogeographica kohnii*, support these predictions. Heart rates of embryos and hatchlings were similar at low temperatures, but heart rates at higher temperatures were much greater before than after hatching.

## Introduction

Hatching (or birth, in viviparous species) entails a profound shift in the physiological functioning of any amniote vertebrate. The offspring must make a rapid transition between the embryonic dependence on extra-embryonic membranes for respiration and excretion, to the free-living stage of reliance on organ systems such as lungs, kidneys, sense organs, skeletal muscles, and the like. Unsurprisingly, then, hatching is accompanied by major shifts in the organism's physiology [1]: not only the acquisition of some functions and the loss of others, but also a modification of the norms of reaction that link organismal function to variable external conditions. One such relationship is the rate at which metabolic processes increase with an increase in body temperature. Although a positive relationship between these two variables is predicted by simple physics and chemistry [2], [3], the exact form of the relationship is flexible, and can be modified by developmentally plastic responses such as acclimation [4].

How might we expect the relationship between temperature and metabolic rate (and correlates of metabolic rate, such as developmental rate and cardiovascular functioning [5]) to be affected by the life-history transition from embryo to hatchling? We suggest that embryonic life should favour a steep reaction norm, whereby metabolic rate increases sharply at higher temperatures. This prediction is based on the embryo benefitting from (a) accelerated developmental rate, because earlier hatching typically enhances offspring fitness [6], [7]; and (b) the assured food supply (in the yolk) to fuel development through to hatching [8]. Both of these factors shift abruptly when the egg hatches. Hatchlings face uncertain levels of food availability, and their fitness often may be enhanced by less-than-maximal rates of growth [9]. Thus, we might expect natural selection to fine-tune thermal norms of reaction, such that higher temperatures induce less of an increase in hatchling metabolic (and thus, heart beat) rates in free-living animals than was the case prior to hatching. In the current paper, we test this prediction with data on heart rates relative to temperature in embryos and hatchlings of two species of emydid turtles.

## Materials and Methods

### Study Species

*Chrysemys picta,*and *Graptemys pseudogeographica kohnii* are two North American freshwater turtles belonging to the family Emydidae. Both species live in ponds, lakes, marshes, and slow-moving rivers [10]. Female *C. picta* lay 4 to 15 oval, soft shelled eggs in shallow nests with mean temperatures from 24 to 28°C [11], and the hatchlings typically spend the first winter of their life in the nests [10]. Both *C. picta* and *G. pseudogeographica kohnii* can elevate their body temperatures above water temperature by basking [12], [13]. Shell temperatures of *C. picta* can vary from 15 to 35°C during the active season from April to October [12].

### Egg Incubation and Measurement of Heart Rate

In May, 2009, we collected freshly-laid eggs of two turtle species, *C. picta*, and *G. pseudogeographica kohnii*, from a private farm in Hankou, Hannan province of China. All eggs were weighed (±0.001 g), and individually incubated at 28°C in 120 ml glass jars filled with moist vermiculite (−200 KPa). Heart rates were measured using an infrared heart rate monitor (Buddy system; Avian Biotech; for detailed methods see [5]). High positive correlations between heart rates and metabolic rates both in embryos [14], [15] and post-hatching individuals [16] mean that heart rates can serve as an index of metabolic rate. The heart rates of embryos were measured twice during incubation, at approximately 25% and 75% through the total incubation period, and the heart rates of hatchlings were measured on the first and tenth day after hatching. To ensure test conditions were similar between embryos and hatchlings, we placed hatchlings inside perforated egg-shaped plastic balls to measure their heart rates. Eggs and hatchlings were allowed to acclimate to test temperatures for two hours (inside incubators set at 20, 25, 30, or 33.5°C), and were then placed individually on the monitor to record heart rate (which generally required less than a minute). The order of exposure of each egg or hatchling to test temperatures was random. Heart rates were repeatedly measured on the same samples of *C. picta* (n = 16),and *G. pseudogeographica kohnii* (n = 15) from eggs to hatchlings. Egg collection and experimental procedures were approved by the Animal Care and Ethics Committee of Hangzhou Normal University and were conducted in accordance with the NIH *Guide for the Principles of Animal Care*.

Repeated measures ANOVAs with species and life-history stage as factors and test temperatures as the repeated variable were conducted to test for ontogenetic shifts and between-species differences in heart rate at different environmental temperatures. One-way ANOVA and Tukey's post-hoc tests were then used to compare heart rate among different life-history stages at each test temperature of each species.

## Results

**Figure 1:**
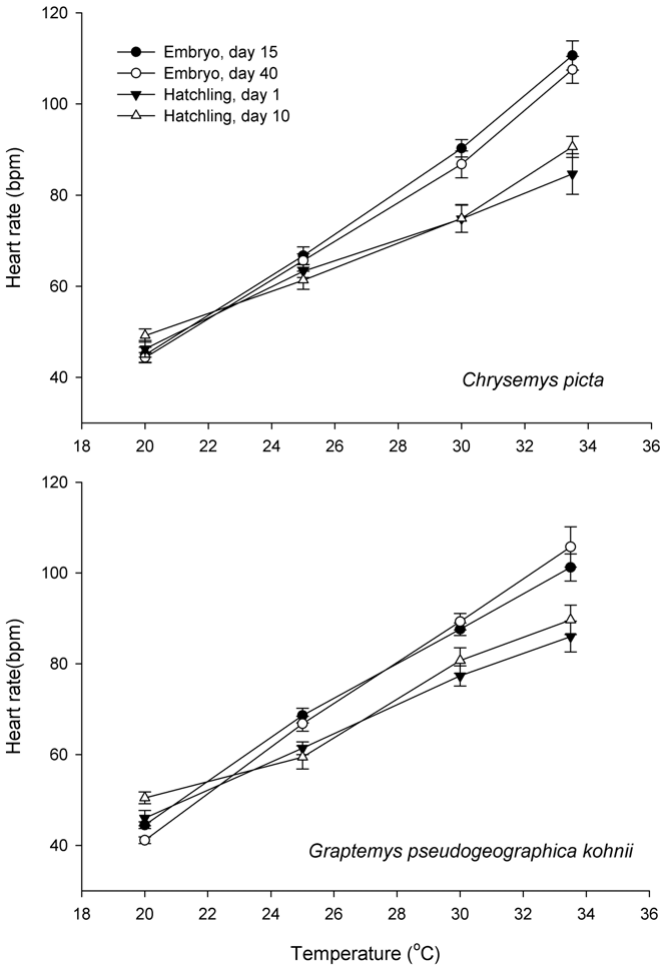
Thermal dependence of heart rates of embryos and hatchlings in the turtles *Chrysemys picta* (a) and *Graptemys pseudogeographica kohnii* (b). Heart rates were measured by non-invasive methods, and are expressed as mean ± standard error. Heart rates did not change with the developmental stage of embryos or with the age of hatchlings, but were greater for embryos than for hatchlings when the animals were tested at high temperatures. Specifically, Turkey's test indicated that significant differences occurred at 30 and 33°C in *C. picta*, and 25, 30 and 33.5°C in *G. pseudogeographica kohnii*.

Eggs of both species (*C. picta* and *G. pseudogeographica kohnii*) hatched after mean incubation periods of 57 and 66 days, respectively. In an analysis including data for both embryos and hatchlings of the two species of turtles, heart rate was significantly affected by test temperature (*F*_3,348_ = 813.9, *P*<0.00001), and changed ontogenetically (*F*_3,116_ = 20.0, *P*<0.00001), but did not differ significantly between species (*F*_1,116_ = 0.12, *P* = 0.73). Heart rates did not differ between successive developmental stages either in embryos or hatchlings, but differed between embryos and hatchlings, with lower heart rates at high temperatures for hatchlings than for embryos (Fig. 1). Q_10_ values of heart rate varied from 1.5 to 2.6 in embryos and from 1.4 to 1.9 in hatchlings depending on temperatures, and differed between embryos and hatchlings, with higher Q_10_ values in embryos (Table 1).

**Table 1:** Q_10_ values of heart rate in different life-history stages in two species of turtles, *Chrysemys picta* and *Graptemys pseudogeographica kohnii*.

	20–25°C	25–30°C	30–33.5°C
*Chrysemys picta*			
Embryo, Day 15	2.20	1.83	1.79
Embryo, Day 40	2.19	1.74	1.85
Hatchling, Day 1	1.87	1.40	1.42
Hatchling, Day 10	1.55	1.49	1.72
*Graptemys pseudogeographica kohnii*			
Embryo, Day 15	2.38	1.63	1.51
Embryo, Day 40	2.64	1.78	1.62
Hatchling, Day 1	1.78	1.58	1.35
Hatchling, Day 10	1.39	1.84	1.35

In most cases, Q_10_ values of heart rate in embryos were higher than those of hatchlings.

## Discussion

The two species of turtles that we studied exhibited similar ontogenetic shifts in heart rate. At the lowest temperature we tested (20°C), heart rates were unchanged from the embryo through to the hatchling stage. At higher temperatures, however, hatchlings exhibited slower heart rates than did embryos (by about 30% at 30°C: see Fig. 1). Because we sampled at only four stages during ontogeny, we cannot identify the exact timing of this developmental shift except to say that it occurred late in embryogenesis (i.e., in the final 25% of the incubation period). Oxygen consumption (and hence energy expenditure) decreases during the last few weeks of incubation in turtles due to rapid decrease of the rate of new tissue synthesis [17]. Heart rates relative to temperature were similar in early and late-stage embryos in our study, suggesting that a decrease might occur close to the time of hatching, and be maintained in post-hatching life. The mechanisms responsible for the ontogenetic shift in heart rates are unknown, but may relate to maturation of cardiovascular control mechanisms in late-term embryos, including both hormonal and nervous regulatory systems [18].

Limited data on other species suggest considerable diversity in the ontogenetic progression of reaction norms linking heart rates to temperature. As in the two turtle species that we studied, embryos of European pond turtles (*Emys orbicularis*) and common snapping turtles (*Chelydra serpentina*) reduce heart rates shortly before hatching [19], [20] as well as after hatching [21]. The low post-hatching heart rates appear to be maintained through into adult life; for example, heart rates of adult *Trachemys scripta*, *Terrapene ornate* and *Gopherus agassizii* are only about 50, 57 and 30 beats/min at 30°C [22], [23], [24]. Thus, shifts in reaction norms linking heart rates to temperature appear to be widespread in turtles. Squamate reptiles show a different pattern, with little or no ontogenetic shift in heart rates from embryo to hatchling stages (e.g., the skink *Bassiana duperreyi* - [25]). Q_10_ coefficients of eastern fence lizards (*Sceloporus undulatus*) are similar between embryos and hatchlings both for metabolic rate (2.1 vs 2.4) [26], [27] and heart rate (2.4 vs 2.3) [28]. In contrast, most bird embryos exhibit an increase in heart rates around the time of hatching [29].

Why do turtles show a decrease in heart rates at hatching, squamates show no shift, and birds show an increase? The data on birds are explained by the development of endothermic metabolism, and hence an elevation in metabolic rate [29]. Our data on turtles are consistent with the hypothesis outlined in the Introduction to this paper. That is, shifts in the benefits *versus* costs of high metabolic rates (and thus, heart rates) between the egg stage and the free-living juvenile may impose selection for adjustment of cardiovascular function. Metabolic rates at low temperatures probably have little impact on fitness in either life-history stage, because energy-expenditure costs are low and little embryogenesis is accomplished at such temperatures [24], [30]. In contrast, metabolic rates at high temperatures likely are under more intense selection for fine-tuning relative to the organism's life-history stage, because both the costs (in energy expenditure) and potential benefits to the embryo but not the hatchling (i.e., rapid embryogenesis) are high. Moreover, in hatchling *C. picta* that overwinter in shallow nests, low heart rates at higher temperatures could also reflect a preparation for low-temperature hibernation, as reported in adult turtles[31].

If turtles benefit by reducing heart rates at hatching, why don't lizards show the same pattern? Annual survival rates (and thus, longevities) average much higher in turtles than in squamates, in both juvenile and adult life-history stages [32]. As a result, short-lived lizards may adopt “riskier” life-history tactics, expending energy at high rates despite the uncertainty about future food availability [9]. The wide range in adult survival rates in squamates [32] provides abundant opportunity to test this explanation, because it predicts decreased heart rates at hatching in long-lived but not short-lived species. Unfortunately, we have so little data that generalizations may be premature.

All oviparous reptiles produce eggs with sufficient yolk to support embryogenesis to the hatchling stage; indeed, a portion of that initially-allocated energy (residual yolk) often is used for maintenance of hatchlings during early post-hatching life [8], [33]. Because embryos have sufficient energy to complete development, and are likely to benefit from early hatching [6], [7], selection should favour adaptations that result in more rapid development within the egg. Given the embryo's inability to thermoregulate behaviourally, increasing metabolic (and thus, developmental) rate at high temperature is probably the most important mechanism available to accelerate embryonic development and growth. Retaining such high metabolic rates post-hatching may enhance offspring fitness in some species (especially, short-lived taxa or those encountereing abundant food), but for many post-hatching reptiles food availability is limited, and may vary unpredictably. Under conditions of low food supply, many reptiles select lower body temperatures, thereby reducing maintenance metabolic costs [34], [35]. A reduction in the slope of the reaction norm linking metabolic rate to temperature (Fig. 1) would result in a similar energy saving. Lifespan is correlated with a fixed [species-specific] number of heart beats in birds and mammals, with lower heart rates in species showing higher longevities [10]. The low heart rate of turtle hatchlings thus may be related to the higher longevities of these species.

## References

[1] Pough EH, Andrews RM, Cadle JE, Crump ML, Savitzky AH (1998). Herpetology..

[2] Gillooly JF, Brown JH, West GB, Savage VM, Charnov EL (2001). Effects of size and temperature on metabolic rate.. Science.

[3] Seebacher F, Franklin CE (2005). Physiological mechanisms of thermoregulation in reptiles: a review.. Journal of Comparative Physiology B.

[4] Huey RB, Berrigan D, Johnston IA, Bennett AF (1996). Testing evolutionary hypotheses of acclimation.. Animals and Temperature.

[5] Du WG, Radder RS, Sun B, Shine R (2009). Determinants of incubation period: do reptilian embryos hatch after a fixed total number of heart beats?. Journal of Experimental Biology.

[6] Warner DA, Shine R (2007). Fitness of juvenile lizards depends on seasonal timing of hatching, not offspring body size.. Oecologia.

[7] Bobyn ML, Brooks RJ (1994). Incubation conditions as potential factors limiting the northern distribution of snapping turtles, *Chelydra serpentina*.. Canadian Journal of Zoology.

[8] Nagle RD, Burke VJ, Congdon JD (1998). Egg components and hatchling lipid reserves: Parental investment in kinosternid turtles from the southeastern United States.. Comparative Biochemistry and Physiology B.

[9] Olsson M, Shine R (2002). Growth to death in lizards.. Evolution.

[10] Ernst CH, Lovich JE (2009). Turtles of the United States and Canada. Baltimore Johns Hopkins University Press.

[11] Morjan CL (2003). Variation in nesting patterns affecting nest temperatures in two populations of painted turtles (*Chrysemys picta)* with temperature-dependent sex determination.. Behavioral Ecology and Sociobiology.

[12] Grayson KL, Dorcas ME (2004). Seasonal temperature variation in the painted turtle (*Chrysemys picta*).. Herpetologica.

[13] Coleman JL, Gutberlet RL (2008). Seasonal variation in basking in two syntopic species of map turtles (Emydidae: *Graptemys*).. Chelonian Conservation and Biology.

[14] Ar A, Tazawa H (1999). Analysis of heart rate in developing bird embryos: effects of developmental mode and mass.. Comparative Biochemistry and Physiology A.

[15] Tazawa H (2005). Cardiac rhythms in avian embryos and hatchlings.. Avian and Poultry Biology Reviews.

[16] Clark TD, Butler PJ, Frappell PB (2006). Factors influencing the prediction of metabolic rate in a reptile.. Functional Ecology.

[17] Booth DT (2000). Incubation of eggs of the Australian broad-shelled turtle, *Chelodina expansa* (Testudinata: Chelidae), at different temperatures: effects on pattern of oxygen consumption and hatchling morphology.. Australian Journal of Zoology.

[18] Crossley DA, Bagatto BR, Dzialowski EM, Burggren WW (2003). Maturation of cardiovascular control mechanisms in the embryonic emu (*Dromiceius novaehollandiae*).. Journal of Experimental Biology.

[19] Nechaeva MV, Vladimirova IG, Alekseeva TA (2007). Oxygen consumption as related to the development of the extraembryonic membranes and cardiovascular system in the European pond turtle (*Emys orbicularis*) embryogenesis.. Comparative Biochemistry and Physiology A.

[20] Birchard GF (2000). An ontogenetic shift in the response of heart rates to temperature in the developing snapping turtle (*Chelydra serpentina*).. Journal of Thermal Biology.

[21] Birchard GF, Reiber CL (1996). Heart rate during development in the turtle embryo: Effect of temperature.. Journal of Comparative Physiology B.

[22] Galli G, Taylor EW, Wang T (2004). The cardiovascular responses of the freshwater turtle *Trachemys scripta* to warming and cooling.. Journal of Experimental Biology.

[23] Voigt WG (1975). Heating and cooling rates and their effects upon heart rate and subcutaneous temperatures in desert tortoise, *Gopherus agassizii*.. Comparative Biochemistry and Physiology A.

[24] Gatten RE (1974). Effects of temperature and activity on aerobic and anaerobic metabolism and heart rate in turtles *Pseudemys scripta* and *Terrapene ornata*.. Comparative Biochemistry and Physiology A.

[25] Radder R, Shine R (2006). Thermally induced torpor in fullterm lizard embryos synchronizes hatching with ambient conditions.. Biology Letters.

[26] Angilletta MJ (2001). Thermal and physiological constraints on energy assimilation in a widespread lizard (*Sceloporus undulatus*).. Ecology.

[27] Angilletta MJ, Lee V, Silva AC (2006). Energetics of lizard embryos are not canalized by thermal acclimation.. Physiological and Biochemical Zoology.

[28] Dzialowski EM, O'Connor MP (2001). Physiological control of warming and cooling during simulated shuttling and basking in lizards.. Physiological and Biochemical Zoology.

[29] Pearson JT, Tazawa H (1999). Ontogeny of heart rate in embryonic and nestling crows (*Corvus corone* and *Corvus macrorhynchos*).. Journal of Comparative Physiology B.

[30] Ewert MA, Gans C, Billett F, Maderson PFA (1985). Embryology of turtles.. Biology of the Reptilia.

[31] Overgaard J, Gesser H, Wang T (2007). Tribute to P. L. Lutz: cardiac performance and cardiovascular regulation during anoxia/hypoxia in freshwater turtles.. Journal of Experimental Biology.

[32] Pike DA, Pizzatto L, Pike BA, Shine R (2008). Estimating survival rates of uncatchable animals: The myth of high juvenile mortality in reptiles.. Ecology.

[33] Ji X, Sun PY (2000). Embryonic use of energy and post-hatching yolk in the gray rat snake, *Ptyas korros* (Colubridae).. Herpetological Journal.

[34] Brown RP, Griffin S (2005). Lower selected body temperatures after food deprivation in the lizard *Anolis carolinesis*.. Journal of Thermal Biology.

[35] Angilletta MJ (2009). Thermal Adaptation: A Theoretical and Empirical Synthesis..

